# The development of metacognitive ability in adolescence

**DOI:** 10.1016/j.concog.2013.01.004

**Published:** 2013-03

**Authors:** Leonora G. Weil, Stephen M. Fleming, Iroise Dumontheil, Emma J. Kilford, Rimona S. Weil, Geraint Rees, Raymond J. Dolan, Sarah-Jayne Blakemore

**Affiliations:** a Institute of Cognitive Neuroscience, University College London, UK; b Wellcome Trust Centre for Neuroimaging, University College London, UK; c Center for Neural Science, New York University, USA; d Department of Experimental Psychology, University of Oxford, UK

**Keywords:** Adolescence, Metacognition, Cognitive development, Self-awareness, Introspection

## Abstract

Introspection, or metacognition, is the capacity to reflect on our own thoughts and behaviours. Here, we investigated how one specific metacognitive ability (the relationship between task performance and confidence) develops in adolescence, a period of life associated with the emergence of self-concept and enhanced self-awareness. We employed a task that dissociates objective performance on a visual task from metacognitive ability in a group of 56 participants aged between 11 and 41 years. Metacognitive ability improved significantly with age during adolescence, was highest in late adolescence and plateaued going into adulthood. Our results suggest that awareness of one’s own perceptual decisions shows a prolonged developmental trajectory during adolescence.

## Highlights

► Metacognition refers to the knowledge we have of our own cognitive processes. ► We investigated the development of metacognition between 11 and 41 years. ► Participants carried out a visual decision task and rated confidence in their decisions. ► While task performance was stable, metacognition improved between 11 and 17. ► Metacognition shows a prolonged developmental trajectory during adolescence.

## 1 Introduction

The ability to reflect upon our own thoughts and behaviour, known as metacognition or introspection, pervades many aspects of experience (Metcalfe, 1996), and is particularly well developed in humans (see Frith (2012), Smith, Shields, and Washburn (2003), Terrace and Son (2009) for discussion of comparative studies). Early research on metacognition distinguished between metacognitive knowledge (knowledge about our own and other people’s cognitive processes) and metacognitive experiences (conscious cognitive or affective experiences that accompany current behaviour; Flavell, 1979). Subsequently, an additional “monitoring” component was proposed, corresponding to the use of metacognitive knowledge and experiences to guide behaviour (Nelson & Narens, 1990). This monitoring process is linked to self-regulation and executive control skills associated with prefrontal cortex (Fernandez-Duque, Baird, & Posner, 2000; Fleming & Dolan, 2012; Frith, 2012; Schneider, 2008; Shimamura, 2000). Research in the field of executive functions has mainly focused on a more implicit system of conflict and error monitoring supported by the posterior medial prefrontal cortex (PFC) (e.g. Ridderinkhof, van den Wildenberg, Segalowitz, & Carter, 2004), rather than the explicit monitoring and control associated with metacognition (see Fleming and Dolan (2012) for a review).

Metacognition is important in decision-making. For example, a meta-level of modulation and coordination between memory retrieval and problem-solving processes is involved in the generation of plans and the evaluation of options, in particular in situations where the solution is not obvious (e.g. Zysset, Huber, Ferstl, & von Cramon, 2002), or when decisions are made jointly between two people (Bahrami et al., 2010; Frith, 2012). More generally, every decision is associated with a degree of confidence, and assessments of confidence can be used to guide current and future decisions (see Kepecs and Mainen (2012) for a review). Thus, knowing what we do not know can motivate us to seek out new information (Metcalfe & Finn, 2008) and communicate our uncertainty to others (Bahrami et al., 2010).

A complementary perspective on metacognition is that it is tightly related to theory of mind (also referred to as mentalising or mindreading), the ability to attribute mental states to other people (Carruthers, 2009; Efklides, 2008; Kuhn, 2000; Schneider, 2008). One view is that mentalising and metacognition represent two different kinds of access to one metarepresentational faculty: mentalising involves the perception of others’ behaviour; metacognition involves introspecting about one’s own behaviour (Frith & Happe, 1999). Another view is that the attribution of mental states to others depends on inference about, or simulation of, one’s own mental states, so introspection about one’s own mental states occurs developmentally prior to mentalising about other people (Goldman, 2006). A third proposal is that introspection about one’s own mental states involves turning mindreading capacities to one’s own behaviour. Unlike other theories, which assume that mentalising and metacognition involve different mechanisms, this view assumes that mentalising and metacognition involve identical mechanisms and inputs (perception and inner speech respectively; Carruthers, 2009; Schneider, 2008). Finally, other theorists consider mentalising about others’ mental states and metacognition to be independent processes (Nichols & Stich, 2003).

In the current study, we investigated the development of metacognitive ability for performance during a perceptual task during adolescence. Specifically, we tested the relationship between confidence and task performance across development, which we refer to as metacognitive ability (although note that we do not test how this corresponds to other forms of metacognition or mentalising). We hypothesised that metacognitive ability would show developmental changes during adolescence, a period of life characterised by changes in mentalising (Dumontheil, Apperly, & Blakemore, 2010), the emergence of self-identity (Sebastian, Burnett, & Blakemore, 2008), and maturation of online performance monitoring visible in particular in response inhibition tasks (Luna, Padmanabhan, & O’Hearn, 2010). There are changes during early and late childhood for several aspects of metamemory, including improvements in the estimation of memory ability and increased use of strategies (Ghetti, Castelli, & Lyons, 2010; Karably & Zabrucky, 2009). Some studies investigating the development of metacognitive monitoring have shown that confidence judgements about memory retrieval of individual items, and more specifically uncertainty monitoring, improve during late childhood (age 7–12 years; Ghetti, Lyons, Lazzarin, & Cornoldi, 2008; Krebs & Roebers, 2010; Roderer & Roebers, 2010; von der Linden & Roebers, 2006). During adulthood, metamemory skills decrease between young (20s) and older ages (70s) (Souchay & Isingrini, 2004). A second area of developmental research has linked the self-regulation aspect of metacognition abilities, in particular the processes of monitoring and control, to executive functions and their development (Fernandez-Duque et al., 2000; Lyons & Zelazo, 2011; Schneider, 2008; Shimamura, 2000). Fewer studies have investigated the development of metacognition following performance on experimental tasks during adolescence. In one study, adolescents (aged 13–15 years) and adults evaluated their performance on propositional, spatial and social reasoning tasks and self-evaluation improved between adolescence and adulthood (Demetriou & Bakracevic, 2009).

Importantly, previous developmental studies of metacognitive ability have not employed tasks in which task performance can be dissociated from metacognitive judgments, a critical issue when studying development. Indeed, a central methodological problem with studying any metacognitive process arises from the tight relationship between awareness and performance (Galvin, Podd, Drga, & Whitmore, 2003). In other words, when a participant knows the answer to a question, they tend to know they know the answer. Recently, we described a psychophysical procedure to dissociate objective performance from adult participants’ evaluation of their performance (Fleming, Weil, Nagy, Dolan, & Rees, 2010). Using this approach, together with structural Magnetic Resonance Imaging (MRI), metacognitive ability in adults, defined here as how accurately participants’ confidence in their performance tracks their actual performance, was shown to correlate positively with grey matter volume in the right rostrolateral prefrontal cortex (RLPFC or Brodmann area 10). This brain region undergoes protracted structural and functional development during adolescence (Giedd et al., 1999; Giedd & Rapoport, 2010; Gogtay et al., 2004). In the current study, we employed a similar behavioural paradigm to characterise metacognitive ability independently from objective performance in a new sample of adolescents and adults (aged between 11 and 41 years) in a visual perceptual task. Our aim was to examine how metacognitive ability changes during development. Based on previous studies of metamemory and mentalising in adolescence, we predicted that metacognitive ability on this perceptual task would show developmental change during this period of life.

## 2 Materials and methods

### 2.1 Participants

Data from 28 healthy adults (10 males; age range 19–41 years; mean age 25.7; SD 4.9), and 28 healthy adolescents (10 males; age range 11–17 years; mean age 14.90; SD 2.00), were included in the analysis. Four additional participants were tested but excluded from the analysis: one had a new epileptic seizure 1 month after testing; the data from one participant were excluded as she never stabilised on the staircase in the perceptual task (see below; Levitt, 1971); and demographic data from two participants were missing. Note that there was no overlap between participants tested in the current study and those tested in our previous study (Fleming et al., 2010). Adult participants gave written consent to participate, while consent was given by the parent/guardian of the adolescent participants. The study was approved by the local Research Ethics Committee.

Participants were recruited through contacting local schools and word of mouth. Potential participants were excluded if they had a history of previous neurological illness, prematurity (<34 weeks gestation), a diagnosis of epilepsy, or any developmental disorder. All participants had normal or corrected-to-normal vision. Participants received £10 for participating.

Each participant (except five adult participants) completed the Vocabulary and Matrix reasoning subtests of the Wechsler Abbreviated Scale of Intelligence (Wechsler, 1999). There was no significant difference between the estimated IQ of adults and adolescents (*t*(49) = −0.29, *p* > .70), and IQ was not correlated with age across the whole sample (Pearson *r* = .13, *p* > .30) or with metacognitive ability (Pearson *r* = .064, *p* > .65).

### 2.2 Experimental design

**Fig. 1:**
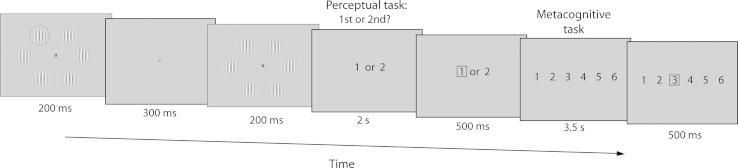
Experimental procedure: Example of the stimulus sequence corresponding to one trial. One of two intervals contained six Gabor patches presented around a fixation point with one ‘pop-out’ Gabor (circled for illustrative purposes). The other interval contained six identical Gabors. Participants were prompted to choose whether the pop-out Gabor was in the 1st or 2nd interval (perceptual task). They were then prompted to rate their confidence in their decision being correct from 1 to 6 (metacognitive task). The task consisted of 350 trials, split into five blocks of 70 trials with four breaks.

The task was computer-based and adapted from our recent metacognition study in adults (Fleming et al., 2010). On each trial, participants performed a perceptual task. The stimuli used were Gabor patches: circular patches of alternating light and dark vertical bars (2.8 visual degrees in diameter, spatial frequency of 2.2 cycles per visual degree). The contrast between the vertical lines in each standard Gabor patch was 20%, where 0% indicates no difference between the light and dark bars and 100%, the maximum difference (black to white). Six such Gabor patches were arranged in a circle (eccentricity of 6.9 visual degrees) around a central fixation point (Fig. 1). One of the six Gabor patches could be made to pop-out from the others by increasing the contrast between the vertical lines in that patch compared with the standard 20% contrast of all the others. The contrast of the pop-out Gabor patches varied from 23% (little effect of pop-out) to 80% (pop-out very clear). The contrast of the starting pop-out stimulus was initially set at 53% and reduced to 50% after noting that the average threshold level after stabilisation was considerably lower than 50% in our first eight participants. The background for the Gabor patches was a uniform grey screen (luminance 3.66 cd/m^2^).

Participants viewed two stimulus arrays each lasting 200 ms, separated by an interval of 300 ms (see Fig. 1). Each array contained the six Gabor patches around a central fixation point, set against the uniform grey background. The interval between stimuli comprised a uniform grey screen without the Gabor patches. A single Gabor patch in one of the two intervals was designated as a pop-out. Which of the six Gabor patches popped-out varied randomly between trials. Participants were prompted by a computer display to respond ‘1’ or ‘2’ (presented centrally; luminance 13.64 cd/m^2^) as to whether they thought the pop-out Gabor patch appeared during the first or second presentation. Participants responded by pressing the numerical keys on the top left-hand side of the laptop keyboard with their left hand. Participants had 2 s in which to make their decision, after which, a red box surrounded their selection. No feedback was given as to whether they were right or wrong.

Participants then indicated confidence in their decision on a scale of 1–6 (1: relatively low confidence; 6: relatively high confidence). The display screen consisted of the numbers 1–6 presented centrally (luminance 13.64 cd/m^2^). Participants were encouraged to use the full range of the scale, thinking carefully about how confident they were after each decision. Participants responded by pressing numerically marked keys on the right-hand side of the laptop keyboard with their right hand, with a red box again surrounding this selection. Participants had 3.5 s to complete this metacognitive judgement.

A standard task instruction sheet explaining the task was read through twice by participants, first on their own, and then together with the task administrator. Participants were seated in a darkened room approximately 60 cm from a laptop computer screen (gamma calibrated Dell Inspiron 1525, 15 in. display; 1280 × 800 pixels). Stimulus display and responses for the tasks were programmed in MATLAB 7.8 (Mathworks Inc., Natick, MA, USA using the COGENT 2000 toolbox (http://www.vislab.ucl.ac.uk/cogent.php)). A practice session of two blocks of eight trials was given at the start to familiarise participants with the task. Participants were tested individually in a quiet room.

### 2.3 Calculating a measure of metacognition

Participants’ overall performance on the perceptual task was maintained at around 71% through use of a 2-down, 1-up staircase procedure (Levitt, 1971), as used previously (Fleming et al., 2010). Two consecutive correct visual judgments led to a one step (3%) decrease in contrast of the pop-out Gabor patch in the next trial, whereas one incorrect visual judgment led to a one step increase in contrast of the pop-out patch. The contrast of the pop-out Gabor patch at the end of each block was used as the starting contrast for the starting pop-out Gabor patch in the next block. Participants were not informed that their performance was being controlled in this way.

Type I *d*′ and bias (*c*) were calculated in the standard manner (Macmillan & Creelman, 2005):

$$d^{\prime }=1/\sqrt{2}[z(H)-z(F)]$$

c=-0.5[z(H)+z(F)]

where *d*′ is a measure of perceptual performance that is unaffected by response bias (whether the participant has an overall tendency to prefer one of the two responses) and where *z* is the inverse of the cumulative normal distribution function, *H* = *p*(response = 1|interval = 1) and *F* = *p*(response = 1|interval = 2). Note that in this task, *d*′ and the participants’ overall accuracy on the perceptual task, which was maintained at around 71%, are tightly related; controlling % correct was sufficient to control *d*′.

Participants’ confidence ratings were used to construct a measure of metacognitive ability through use of type II signal detection theory (SDT; Galvin et al., 2003). Type II SDT quantifies how well an observer’s metacognitive confidence tracks their objective accuracy on a task. The first 100 trials were excluded from this analysis to permit stabilisation of the staircase, as done previously (Fleming et al., 2010). To plot the receiver operating characteristic (ROC) curve, *h_i_* = *p*(confidence = *i* | correct) and *f_i_* = *p*(confidence = *i* | incorrect) were calculated for all *i*. These probabilities were then transformed into cumulative probabilities, and plotted against each other, producing ROCs anchored at [0, 0] to obtain seven levels of *i* (from the 1 to 6 confidence rating scale, plus the zero point). Following Kornbrot (2006), we computed distribution-free measures of sensitivity and bias from this ROC by dividing the area into two parts – *K_B_* is the area between the ROC curve and the major diagonal above the middle confidence rating, and *K_A_* is the area between the receiver operating characteristic (ROC) curve and major diagonal below the middle confidence rating. From simple geometry [derived in the Appendix of Kornbrot (2006)], these areas are calculated as follows:

$$K_{A}=\frac{1}{4}\overset{k=3}{\underset{k=1}{\sum }}\left[{\left(h_{k+1}-f_{k}\right)}^{2}-{\left(h_{k}-f_{k+1}\right)}^{2}\right]$$

$$K_{B}=\frac{1}{4}\overset{k=6}{\underset{k=4}{\sum }}\left[{\left(h_{k+1}-f_{k}\right)}^{2}-{\left(h_{k}-f_{k+1}\right)}^{2}\right]$$

Sensitivity (*A_roc_*) is then the sum of these areas, and Type II bias (*B_roc_*) is the log of the ratio of these areas:

$$A_{\mathrm{roc}}=K_{A}+K_{B}+0.5$$

$$B_{\mathrm{roc}}=\ln\left(\frac{K_{A}}{K_{B}}\right)$$

### 2.4 Statistical analysis of age effects

Once *A_roc_* and *B_roc_* were estimated for each participant, we first investigated possible correlations between these measures and performance (task difficulty and *d*′) using Pearson’s parametric correlations. We then investigated age effects on both metacognitive and performance measures. In order to allow for both linear changes in metacognitive abilities with age, and also age effects limited to the adolescent group, we performed univariate ANOVAs entering *A_roc_* (or *B_roc_*) as dependent variables, and age group and age as independent variables. Significant age group × age interactions were followed up by performing Pearson’s parametric correlations between *A_roc_* or *B_roc_* and age within each age group. In addition, we tested for potential gender effects on *A_roc_* by comparing male and female participants using an independent *t*-test, as well as by including sex as a predictor in the univariate ANOVA described above.

## 3 Results

### 3.1 Perceptual and metacognitive tasks

Metacognitive ability (*A_roc_*) was calculated from the area under the type II ROC curve for each participant (Fleming et al., 2010; Galvin et al., 2003), which quantifies how closely confidence ratings tracked objective performance. The signal detection model provided a good fit to the confidence rating data (mean *R*^2^ = 0.98 ± 0.02 SD). Metacognitive ability (*A_roc_*) ranged between 0.60 and 0.80 (where 0.5 corresponds to no relationship between confidence and actual performance), despite task performance being held at around 71% (mean 70.9%, SD 1.2%) by adjusting stimulus contrast through the use of the 2-down 1-up staircase procedure (Levitt, 1971) described above.

There was no significant correlation between *A_roc_* and task difficulty (mean stimulus contrast; Pearson’s *r* = −.09, *p* = .50) or *A_roc_* and *d*′ (*r* = .21, *p* = .12).

*B_roc,_*, a measure of the tendency to use higher or lower confidence ratings, was similarly analysed. *B_roc_* ranged between −5.68 and 1.94 (values < 0 corresponding to a bias towards lower confidence ratings, and values > 0 corresponding to a bias towards higher confidence ratings) and showed no correlation with task difficulty (mean stimulus contrast; *r* = −.05, *p* = .70), or *d*′ (*r* = .10, *p* = .45). Note, however, that the confidence scale here is relative, not absolute (i.e. the units do not indicate over- or under-confidence), and thus *B_roc_* results should be interpreted with caution.

### 3.2 Metacognitive ability and age

We performed a univariate ANOVA entering *A_roc_* as the dependent variable and age and age group (adolescent, adult) as independent variables on the whole sample (*N* = 56). This revealed a main effect of age group (*F*_(1,52)_ = 5.04, *p* = .029), with better *A_roc_* in the adolescents than the adults, and a significant age × age group interaction (*F*_(1,52)_ = 6.11, *p* = .017). The main effect of age was not significant (*F*_(1,52)_ = 2.77, *p* > .1).

**Fig. 2:**
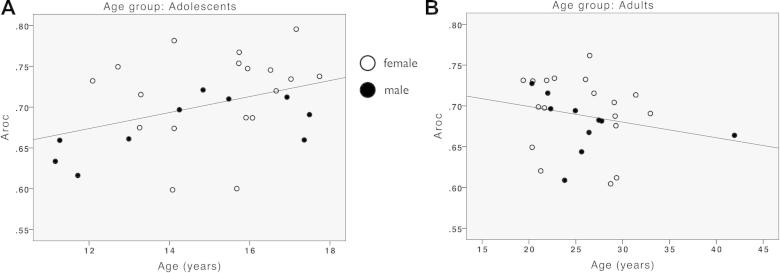
Relationship between *A_roc_*, age and sex. (A) Scatterplot illustrating the significant positive correlation between *A_roc_* and age in adolescence (*r* = .38, *p* = .048). (B) Scatterplot illustrating the non-significant relationship between *A_roc_* and age in adulthood (*r* = −.22, *p* = .25).

The interaction was driven by a significant positive correlation between age and *A_roc_* in the adolescent group (*r* = .38, *p* = .048) (Fig. 2A) and a non-significant but negative correlation in the adult group (*r* = −.22, *p* = .25) (Fig. 2B). Thus metacognitive ability increased during adolescence and remained stable in adulthood. Importantly, parallel analyses showed that psychophysical threshold (mean contrast) and *d*′ were not correlated with age across the whole sample (respectively *r* = −.15, *p* = .27; *r* = −.03, *p* = .80), nor within the adolescent group (respectively *r* = −.30, *p* = .13; *r* = −.19, *p* = .34).

We found that females tended to have greater *A_roc_* than males (females, mean 0.71, SD 0.051; males, mean 0.68, SD 0.034; *t*(54) = 2.2, *p* = .032). However, including a main effect of sex in the univariate ANOVA did not alter the significance of the age × age group interaction (*F*_(1,52)_ = 4.82, *p* = .033). Note that one of the participants was an outlier in terms of age compared to the whole group of participants. Excluding this participant from the analyses did not change the conclusions.

We performed a similar univariate ANOVA of *B_roc_* using age and age group as independent variables. There was no effect of age overall (*F*_(1,52)_ = 3.18, *p* > .8), but a significant main effect of age group (*F*_(1,52)_ = 10.50, *p* = .002) with higher *B_roc_* in adolescents than adults, and an age × age group interaction (*F*_(1,52)_ = 11.5, *p* = .001).

In this case, the interaction effect was driven by a positive correlation between age and *B_roc_* in the adolescents (*r* = .41, *p* = .028), and a negative correlation in the adults (*r* = −.49, *p* = .008). Thus the ratings tended to be biased towards greater confidence over the course of adolescence and declined in adulthood. Note that including *B_roc_* as a covariate in the *A_roc_* analysis does not affect the findings (age group: *F*_(1,52)_ = 3.66, *p* = .061; age: *F*_(1,52)_ = 2.35, *p* > .13; age × age group: *F*_(1,52)_ = 4.39, *p* = .041).

Mean reaction times to both the perceptual and metacognitive judgments did not correlate with age in the whole sample (perceptual: *r* = .16, *p* = .25; metacognitive: *r* = .084, *p* = .54) or within the adolescent group (perceptual: *r* = .20, *p* = .30; metacognitive: *r* = .10, *p* = .60). There was a trend for RTs to positively correlate with *A_roc_* (perceptual: *r* = .28, *p* = .036; metacognitive: *r* = .24, *p* = .074).

## 4 Discussion

In this study, we investigated the development of metacognitive ability from adolescence into adulthood using a psychophysical procedure that dissociated metacognitive ability from task performance. Overall, there was an interaction between age group and metacognitive ability, such that metacognitive ability (*A_roc_*) increased with age during adolescence and plateaued in adulthood. These changes in metacognitive ability were independent from potential age-related changes in perceptual task performance or in confidence bias (*B_roc_*). Although our study was not designed to investigate gender differences, we also found that *A_roc_* varied across sex, with better metacognitive performance in females. This relationship between *A_roc_* and sex was not observed in our previous study, and future studies should be designed to investigate potential sex differences in more detail with larger samples of male and female participants of all ages.

A number of previous studies have examined the capacity for metacognition about one’s performance during adolescence. Self-evaluation improves between adolescence and adulthood on propositional, spatial and social reasoning tasks (Demetriou & Bakracevic, 2009). Similarly, “feeling of knowing” accuracy improves with age, indicating more accurate monitoring (Wellman, 1978). Metacognitive ability in paired-associated learning is impaired in 6-year-old children (due to a failure of error-monitoring) but reaches ceiling by age 10 and remains high in college-age students (age 19; Bisanz, Vesonder, & Voss, 1978). However, younger children (age 6) are better than older children (age 10) and adolescents (age 18) at knowing when they know, despite having poorer memory performance (Butterfield, Nelson, & Peck, 1988), and young adults are better at sensing discrepancies in the control of action (Metcalfe, Teal, & Alan, 2010) than both younger children (8–10 year olds) and older adults (mean age 75).

Notably, these earlier studies did not independently control performance, making it difficult to disentangle variation in metacognitive ability from variation in task performance *per se*. Such control is important to discount a first-order interpretation of variations in metacognitive ability (Fleming, Dolan, & Frith, 2012). On first-order accounts, variability in confidence ratings should be entirely accounted for by changes in primary task performance (Galvin et al., 2003). By clamping variability in task performance using a psychophysical staircase, we were able to isolate variability in confidence judgments of first-order stimulus evaluations, and examine its developmental profile. This component of metacognitive ability increased during adolescence, despite task performance remaining stable (there was no correlation between contrast or *d*′ and age). In summary, our results indicate that metacognitive ability improves across the period of adolescence, is highest in late adolescence and stabilises in adulthood.

It has been proposed that adolescence represents a period in which the sense of self-identity undergoes profound development (Sebastian et al., 2008). How metacognitive ability as measured in the current task relates to self-awareness and self-identity is not well understood, but the gradual improvement in metacognitive ability across the period of adolescence reported here might relate to increased egocentricity, sense of self and developing self-awareness. Speculatively, this might lead adolescents to become more in tune with their own task performance at a stage when they become more aware of, and place more value on the judgments of others (Sebastian et al., 2008), and when they develop individual identities, separate from their families (Lapsley, 1991). It would be interesting in future studies to investigate the relationship between metacognitive ability for perceptual task performance and self-awareness and self-identity in adolescence.

Our results indicate that metacognitive ability for perceptual task performance was higher overall in the adolescent group compared with the adult group. Good metacognitive ability might permit more accurate monitoring of performance and the capacity to assess our abilities at a crucial stage in development. Linked to this is the possibility that metacognitive ability plays a role in improved learning, enabling us to focus more efficiently on what we still need to learn (Metcalfe & Finn, 2008) at a stage where learning plays a key role in our development. Efklides (2009) argues that metacognitive ability plays an important role in self-regulated learning through three different facets: metacognitive knowledge, which acts as a ‘database’ from which learning can be regulated; metacognitive experience, the affective aspect whereby we know or monitor how well we are learning in order to facilitate future learning; and metacognitive skill – the strategies we use to apply our learning. Further studies are needed to identify such a link between metacognitive ability (operationalised here as the relationship between task performance and confidence) and learning strategy, especially during development, a link that maps onto the distinction between monitoring (knowing what we know) and control (using monitoring to direct our cognitive performance, including learning) (Koriat, 2000).

It has been proposed that metacognition is linked to mentalising (Carruthers, 2009). Previous developmental studies of mentalising have demonstrated an improvement in performance on mentalising tasks across adolescence, similar to the improvement in metacognition observed in the current study. For example, the ability to take into account another person’s perspective to guide appropriate behaviour in a communication task continues to improve throughout adolescence (Dumontheil et al., 2010). Although we did not include a mentalising task in the current study, our findings suggest that mentalising and metacognition follow similar developmental trajectories. On the other hand, unlike in the current study in which adolescents overall showed higher performance than adults, the adolescent group in our mentalising study did not show superior performance on the perspective-taking task than the adult group (Dumontheil et al., 2010). It is difficult to compare the results of these two studies, which involved different samples. Future studies should systematically investigate the precise relationship between mentalising and metacognition during development. In addition, whether the kind of metacognitive judgment made in the current task requires mentalising about the self is not known. This could also be investigated in future studies by including tasks that involve making mental state attributions about the self (Frith & Happe, 1999).

Adolescence is a key stage in human development, incorporating physical, social, hormonal and psychological changes (Lerner & Steinberg, 2004). While a small number of studies have examined how awareness of others’ mental states (mentalising) develops during adolescence, little was known about how awareness of one’s own task performance (metacognition) changes with age in a paradigm in which performance and confidence can be dissociated. In the current study, we show that metacognitive ability improves with age over the course of adolescence. We suggest that a gradually improving ability to be aware of one’s own thoughts and behaviour during this period may confer particular benefits for development including the emergence of high level cognitive competencies.

## References

[b0005] Bahrami B., Olsen K., Latham P.E., Roepstorff A., Rees G., Frith C.D. (2010). Optimally interacting minds. Science.

[b0010] Bisanz G.L., Vesonder G.T., Voss J.F. (1978). Knowledge of one’s own responding and the relation of such knowledge to learning a developmental study. Journal of Experimental Child Psychology.

[b0015] Butterfield E.C., Nelson T.O., Peck V. (1988). Developmental aspects of the feeling of knowing. Developmental Psychology.

[b0020] Carruthers P. (2009). How we know our own minds: The relationship between mindreading and metacognition. Behavioral and Brain Sciences.

[b0025] Demetriou A., Bakracevic K. (2009). Reasoning and self-awareness from adolescence to middle age: Organization and development as a function of education. Learning and Individual Differences.

[b0030] Dumontheil I., Apperly I.A., Blakemore S.J. (2010). Online usage of theory of mind continues to develop in late adolescence. Developmental Science.

[b0035] Efklides A. (2008). Metacognition: Defining its facets and levels of functioning in relation to self-regulation and co-regulation. European Psychologist.

[b0040] Efklides A. (2009). The role of metacognitive experiences in the learning process. Psicothema.

[b0045] Fernandez-Duque D., Baird J.A., Posner M.I. (2000). Executive attention and metacognitive regulation. Consciousness and Cognition.

[b0050] Flavell J.H. (1979). Metacognition and cognitive monitoring: A new area of cognitive-developmental inquiry. American Psychologist.

[b0060] Fleming S.M., Dolan R.J. (2012). The neural basis of accurate metacognition. Philosophical Transactions of the Royal Society of London. Series B, Biological sciences.

[b0065] Fleming S.M., Dolan R.J., Frith C.D. (2012). Metacognition: computation, biology and function. Philosophical Transactions of the Royal Society of London. Series B, Biological Sciences.

[b0055] Fleming S.M., Weil R.S., Nagy Z., Dolan R.J., Rees G. (2010). Relating introspective accuracy to individual differences in brain structure. Science.

[b0070] Frith C.D. (2012). The role of metacognition in human social interactions. Philosophical Transactions of the Royal Society of London. Series B, Biological sciences.

[b0075] Frith U., Happe F. (1999). Theory of mind and self-consciousness: What is it like to be autistic?. Mind and Language.

[b0080] Galvin S., Podd J., Drga V., Whitmore J. (2003). Type 2 tasks in the theory of signal detectability: Discrimination between correct and incorrect decisions. Psychonomic Bulletin & Review.

[b0090] Ghetti S., Castelli P., Lyons K.E. (2010). Knowing about not remembering: Developmental dissociations in lack-of-memory monitoring. Developmental Science.

[b0085] Ghetti S., Lyons K.E., Lazzarin F., Cornoldi C. (2008). The development of metamemory monitoring during retrieval: The case of memory strength and memory absence. Journal of Experimental Child Psychology.

[b0095] Giedd J.N., Blumenthal J., Jeffries N.O., Castellanos F.X., Liu H., Zijdenbos A. (1999). Brain development during childhood and adolescence. a longitudinal MRI study. Nature Neuroscience.

[b0100] Giedd J.N., Rapoport J.L. (2010). Structural MRI of pediatric brain development: What have we learned and where are we going?. Neuron.

[b0105] Gogtay N., Giedd J.N., Lusk L., Hayashi K.M., Greenstein D., Vaituzis A.C. (2004). Dynamic mapping of human cortical development during childhood through early adulthood. Proceedings of the National Academy of Sciences of USA.

[b0110] Goldman A.I. (2006). Simulating minds: The philosophy, psychology, and neuroscience of mindreading.

[b0115] Karably K., Zabrucky K.M. (2009). Children’s metamemory: A review of the literature and implications for the classroom. International Electronic Journal of Elementary Education.

[b0130] Kepecs A., Mainen Z.F. (2012). A computational framework for the study of confidence in humans and animals. Philosophical Transactions of the Royal Society of London. Series B, Biological Sciences.

[b0125] Koriat A. (2000). The feeling of knowing: Some metatheoretical implications for consciousness and control. Consciousness and Cognition.

[b0120] Kornbrot D.E. (2006). Signal detection theory, the approach of choice: Model-based and distribution-free measures and evaluation. Perception & Psychophysics.

[b0135] Krebs S.S., Roebers C.M. (2010). Children’s strategic regulation, metacognitive monitoring, and control processes during test taking. British Journal of Educational Psychology.

[b0140] Kuhn D., Mitchell P., Riggs K.J. (2000). Theory of mind, metacognition, and reasoning: A life-span perspective. Children’s reasoning and the mind.

[b0145] Lapsley D.K., Lerner R.M. (1991). Egocentrism theory and the ‘New Look’ at the imaginary audience and personal fable in adolescence. Encyclopaedia of adolescence.

[b0150] Lerner R.M., Steinberg L. (2004). Handbook of adolescent psychology.

[b0155] Levitt H. (1971). Transformed up–down methods in psychoacoustics. Journal of the Acoustical Society of America.

[b0160] Luna B., Padmanabhan A., O’Hearn K. (2010). What has fMRI told us about the development of cognitive control through adolescence?. Brain and Cognition.

[b0165] Lyons K.E., Zelazo P.D., Benson Janette (2011). Monitoring, metacognition, and executive function: Elucidating the role of self-reflection in the development of self-regulation.

[b0170] Macmillan N.A., Creelman C.D. (2005). Detection theory: A user’s guide.

[b0175] Metcalfe J. (1996). Metacognition: Knowing about knowing.

[b0180] Metcalfe J., Finn B. (2008). Evidence that judgments of learning are causally related to study choice. Psychonomic Bulletin & Review.

[b0185] Metcalfe J., Teal S.E., Alan D.C. (2010). Metacognition of agency across the lifespan. Cognition.

[b0190] Nelson T.O., Narens L., Bower G. (1990). Metamemory: A theoretical framework and new findings.

[b0195] Nichols S., Stich S., Smith Q., Jokic A. (2003). How to read your own mind: A cognitive theory of self-consciousness. Consciousness: New philosophical essays.

[b0200] Ridderinkhof K.R., van den Wildenberg W.P., Segalowitz S.J., Carter C.S. (2004). Neurocognitive mechanisms of cognitive control: The role of prefrontal cortex in action selection, response inhibition, performance monitoring, and reward-based learning. Brain and Cognition.

[b0205] Roderer T., Roebers C.M. (2010). Explicit and implicit confidence judgments and developmental differences in metamemory: An eye-tracking approach. Metacognition and Learning.

[b0210] Schneider W. (2008). The development of metacognitive knowledge in children and adolescents: Major trends and implications for education. Mind, Brain and Education.

[b0215] Sebastian C., Burnett S., Blakemore S.J. (2008). Development of the self-concept during adolescence. Trends in Cognitive Sciences.

[b0220] Shimamura A.P. (2000). Toward a cognitive neuroscience of metacognition. Consciousness and Cognition.

[b0225] Smith J.D., Shields W.E., Washburn D.A. (2003). The comparative psychology of uncertainty monitoring and metacognition. Behavioral and Brain Sciences.

[b0230] Souchay C., Isingrini M. (2004). Age related differences in metacognitive control: Role of executive functioning. Brain and Cognition.

[b0235] Terrace H.S., Son L.K. (2009). Comparative metacognition. Current Opinion in Neurobiology.

[b0240] von der Linden N., Roebers C.M. (2006). Developmental changes in uncertainty monitoring during an event recall task. Metacognition and Learning.

[b0245] Wechsler D. (1999). Wechsler abbreviated scale of intelligence (WASI).

[b0250] Wellman H.M. (1978). Knowledge of the interaction of memory variables: A developmental study of metamemory. Developmental Psychology.

[b0255] Zysset S., Huber O., Ferstl E., von Cramon D.Y. (2002). The anterior frontomedian cortex and evaluative judgment: An fMRI study. NeuroImage.

